# The Role of the Left Inferior Parietal Cortex in Gilles de la Tourette Syndrome—An rTMS Study

**DOI:** 10.3390/biomedicines11030980

**Published:** 2023-03-22

**Authors:** Theresa Paulus, Lynn Wernecke, Annik Lundie, Julia Friedrich, Julius Verrel, Tina Rawish, Anne Weissbach, Christian Frings, Christian Beste, Tobias Bäumer, Alexander Münchau

**Affiliations:** 1 Institute of Systems Motor Science, University of Lübeck, 23562 Lübeck, Germany; 2 Department of Neurology, University of Lübeck, 23562 Lübeck, Germany; 3 Institute of Neurogenetics, University of Lübeck, 23562 Lübeck, Germany; 4 Department of Cognitive Psychology, Faculty of Psychology, University of Trier, 54296 Trier, Germany; 5 Cognitive Neurophysiology, Department of Child and Adolescent Psychiatry, Faculty of Medicine, Technische Universität Dresden, 01307 Dresden, Germany; 6 Neuropsychology Centre, Faculty of Medicine, Technische Universität Dresden, 01307 Dresden, Germany; 7 Cognitive Psychology, Faculty of Psychology, Shandong Normal University, Jinan 250014, China

**Keywords:** Gilles de la Tourette syndrome, tics, repetitive transcranial magnetic stimulation, left inferior parietal cortex, BA40, rush score

## Abstract

Increased activity in the left inferior parietal cortex (BA40) plays a role in the generation of tics in the Gilles de la Tourette syndrome (GTS). Thus, inhibitory repetitive transcranial magnetic stimulation (rTMS) applied to BA40 was hypothesized to alleviate symptoms in GTS. We investigated the immediate effects of single-session 1 Hz rTMS and sham stimulation delivered to the left BA40 on tics assessed with the Rush video protocol in 29 adults with GTS. There were no significant effects on tic symptoms following rTMS or sham stimulation. Moreover, there was no difference when comparing the effects of both stimulation conditions. Bayesian statistics indicated substantial evidence against an intervention effect. The left BA40 appears not to be a useful target for 1 Hz rTMS to modulate tic symptoms in GTS patients.

## 1. Introduction

Gilles de la Tourette syndrome (GTS) is a common neuropsychiatric disorder characterized by the presence of motor and vocal tics [1]. Several studies have investigated the effects of repetitive transcranial magnetic stimulation (rTMS), a widely used but still experimental noninvasive brain stimulation technique [2], on symptom severity in GTS [3,4]. The rationale is that rTMS can lead to longer-lasting excitability changes, i.e., neuroplasticity, in the stimulated cortical region and interconnected brain networks rendering this technique attractive for therapeutic interventions [4]. However, robust evidence for clinical efficacy is as yet scarce.

Previous studies investigating rTMS as a treatment for GTS symptoms, particularly protocols where rTMS is given to the supplementary motor area (SMA), have provided mixed results. Whereas rTMS was effective in reducing tic severity assessed by the Yale Global Tic Severity Scale (YGTSS) in some open-label studies [5,6,7,8], in sham-controlled studies, no significant reduction and strong placebo effects were demonstrated [9,10,11,12]. Stimulation of the SMA appears to be more effective [5,6,7,8,13,14] than stimulation of other brain areas [9,12]. One study reported a significant reduction in tics and premonitory urges in GTS patients after 0.5 Hz bilateral rTMS of the parietal cortex, indicating that this region might also be an attractive target for low-frequency rTMS [15]. In line with this, a functional magnetic resonance imaging (MRI) study found increased activation in parietal areas in periods before tic onset in GTS patients [16]. Moreover, in a study examining perception-action processing in adults with GTS, abnormally increased perception-action binding was related to the occurrence of tics and associated with activation differences in the left inferior parietal cortex, i.e., Brodmann area 40 (BA40) [17]. These findings suggest that BA40 is a promising rTMS target for reducing tic symptoms [4]. Against the background of increased tic-related activation of parietal areas [16] and increased perception-action binding associated with left BA40 activation in GTS [17], inhibitory, low-frequency, i.e., 1 Hz rTMS applied to the left BA40 appears to be an attractive measure to improve tic symptoms. We thus examined the immediate effects of single-blinded, single-session 1 Hz rTMS versus sham stimulation delivered to the left BA40. We hypothesized that rTMS but not sham stimulation would improve symptoms in GTS.

## 2. Materials and Methods

### 2.1. Participants and Clinical Assessment

A total of 29 adults with GTS (16 males, 13 females, mean age 30.28 ± 9.83 SD, range 18–50 years), diagnosed according to DSM-5 criteria [1], were recruited from specialized outpatient clinics of the Institute of Systems Motor Science in cooperation with the Departments of Neurology and of Psychiatry and Psychotherapy at the University Medical Center Schleswig-Holstein, Campus Lübeck, Germany. Exclusion criteria were other neurological diseases, psychosis or a major depressive episode at the time of study participation, pregnancy, an IQ below 80, abnormalities in the MRI, and any contraindication for rTMS or MRI (e.g., metal foreign bodies and metal implants).

All participants underwent a comprehensive clinical assessment as performed previously [17] and completed two rTMS sessions (rTMS and sham stimulation). The clinical assessment included the Mini International Neuropsychiatric Interview [18] to detect psychiatric comorbidities, the Yale-Brown Obsessive Compulsive Scale (Y-BOCS) [19] to assess symptoms of obsessive-compulsive disorder, the German version of the Conners Adult Attention deficit hyperactivity disorder (ADHD) Rating Scale [20] to rate ADHD symptoms, and the short form of the Wechsler Adult Intelligence Scale [21]. Further, the clinical assessment included the Yale Global Tic Severity Scale (YGTSS) [22], the Diagnostic Confidence Index (DCI) [23], and the Premonitory Urge for Tic Scale (PUTS) [24]. To capture the typical fluctuation of symptoms [25], we repeated the YGTSS and PUTS directly before starting the second stimulation session.

To investigate the effects of rTMS on tic symptoms, a standardized ten-minute video of each participant was recorded before and after each stimulation session using the Rush video protocol [26]. We recorded the two body views, “full frontal body” and “head and shoulders”, under the two conditions, “with the examiner in the room” and “the patient alone in the room”. Each video segment lasted 2.5 min. Two experienced, independent raters scored the videos using the well-established Modified Rush Videotape Rating Scale (MRVRS) [26]. This kind of video rating allows precise ratings of motor and vocal tics with respect to severity, frequency, and anatomic distribution of tics [26]. Prior to rating, the videos were anonymized. The raters were blind regarding the experimental condition (rTMS or sham stimulation) and the time of video recording (pre- or post-rTMS). Only video segments with no examiner in the room were scored (5 min).

Rush video analysis included a rating of five categories (number of body areas, frequency of motor tics, frequency of vocal tics, severity of motor tics, and severity of vocal tics) on a scale from 0 to 4. The total tic score ranges from 0 to 20 [26]. In addition, we calculated the motor tic count per minute because a previous study found this parameter to correlate with increased perception-action binding in adults with GTS, which in turn was associated with activation differences in the left BA40 [17].

We calculated the mean values of the two raters when the total score or tic count per minute differed by less than 15%. When these scores differed by more than 15%, relevant video segments were discussed and reviewed to reach a consensus score, and a tic count per minute differed by less than 15% [27]. 

In this study, the effect of rTMS on all Rush variables (number of body areas, frequency of motor tics, frequency of vocal tics, severity of motor tics, severity of vocal tics, and total score) and motor tic count per minute was investigated. The main outcomes were the Rush total score because this variable includes the frequency and severity of both vocal and motor tics and the tic count per minute for a reason outlined above. 

All participants had stable medication for at least two weeks prior to participation. During testing, 11 of 29 patients took medication to treat tics, including aripiprazole (*n* = 2), cannabinoid medications (*n* = 2), pimozide (*n* = 1), olanzapine (*n* = 1), risperidone (*n* = 1), amisulpride (*n* = 1), amphetamine (*n* = 1), and antidepressants (*n* = 2).

The study was performed in accordance with the Declaration of Helsinki and was approved by the local ethics committee (reference number 17–156; date of approval: 1 June 2017). All participants provided written informed consent for study participation.

### 2.2. rTMS Protocol

All participants completed two rTMS sessions (rTMS and sham stimulation) in a pseudo-randomized and counter-balanced order, separated by at least one week to avoid carry-over effects. Participants were blinded to the experimental condition (rTMS or sham stimulation), and rTMS was well-tolerated.

After completion of the clinical assessment, including the first Rush video recording, the participant was seated in a comfortable chair, and the head was placed on a chin rest to minimize head movements and maintain the rTMS coil position during stimulation. Participants were instructed to relax. 

We determined the resting motor threshold (RMT) of the left primary motor cortex for the right first dorsal interosseous muscle in each session for each participant while the right forearm was in a relaxed position [28]. For this procedure, the coil was positioned above the “motor hotspot”. The “motor hotspot” was defined as the optimal coil position where stimuli of slightly supra-threshold intensity consistently produced the largest motor evoked potential (MEP) of the right first dorsal interosseous (FDI) muscle. MEPs were recorded with Ag/Ag-Cl disc surface electrodes placed over the right FDI muscle in a belly-tendon montage. The ground electrode was attached above the wrist. The electromyography signal was amplified and band-pass filtered (20 Hz–2 kHz) using a D360 amplifier (Digitimer Limited, Welwyn Garden City, Hertfordshire, UK), sampled with a rate of 5 kHz (Micro 1401, Cambridge Electronics Design (CED), Cambridge, UK), and stored on a computer using Signal 6.0 software (CED, Cambridge, UK). The RMT was defined as the lowest stimulation intensity that produced an MEP response of 50–100 µV in a minimum of 5 out of 10 consecutive trials in the relaxed FDI.

The target region (left BA40) was identified in each participant by using the individual T1-weighted high-resolution MRI scan that was performed before the first rTMS session on a 3 T MR scanner (Magnetom Skyra, Siemens, Erlangen, Germany). A FLASH 3D sequence (TR = 1900 ms, TE = 2.44 ms, TI = 900 ms, flip angel 9°, 1 × 1 × 1 mm^3^ resolution, 192 × 256 × 256 mm^3^ field of view; acquisition time 4.5 min) was used. We imported MRI scans into the stereotaxic neuronavigation system (Brainsight Rogue Research Inc., Montreal, Quebec, QC, Canada). Based on the MRI scan, the surface of the head and of the brain were exactly calculated, and the left BA40 was placed within the inferior parietal lobule, posterior to the postcentral sulcus, and superior to the lateral sulcus, using published MNI [29] coordinates x = −48, y = −34, z = 36 [30]. The stimulation coil and the head of the participant were registered in three-dimensional space using an optical tracking system (Polaris, NDI Medical Solutions, Ontario, ON, Canada). 

For rTMS, the frequency was 1 Hz, and the stimulation intensity was 120% of the RMT. For the sham stimulation, we used an intensity of 40% RMT (30% of the stimulation intensity of the rTMS condition) [31]. In each test session, 1200 pulses were applied. Repetitive TMS was performed with a Magstim Rapid stimulator and a figure-of-eight coil with an outer diameter of 70 mm. Each magnetic stimulus had a biphasic waveform and a pulse width of about 300 µs. 

Using the Brainsight TMS neuronavigation system, the position and the stability of coil placement were monitored precisely during the entire rTMS and sham stimulation session. 

**Figure 1:**
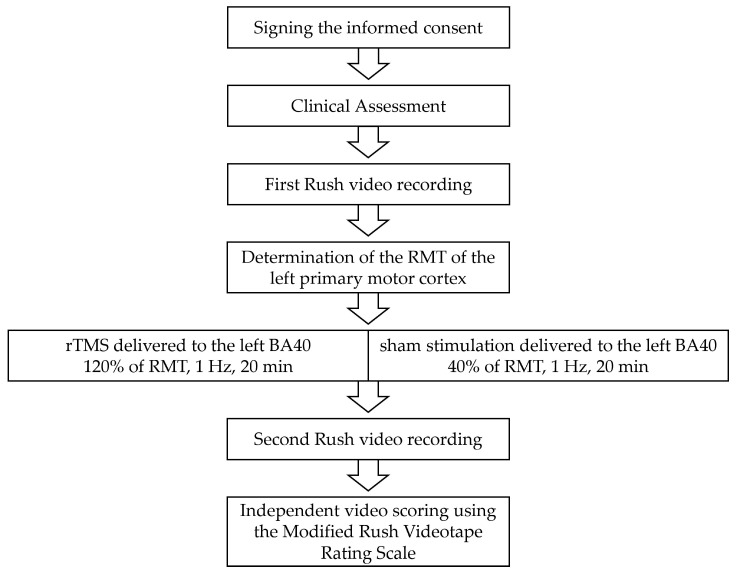
Flow diagram of the study procedure. After obtaining written informed consent, a clinical assessment was conducted. Subsequently, the first Rush video protocol was recorded to capture tic symptoms. Next, following resting motor threshold (RMT) determination, rTMS or sham stimulation was applied. This was followed by the second Rush video protocol recording. Finally, two independent raters scored the videos using the Modified Rush Videotape Rating Scale.

An overview of the study procedure is presented in Figure 1.

### 2.3. Statistical Analysis

All variables of the MRVRS (number of body areas, frequency of motor tics, frequency of vocal tics, severity of motor tics, severity of vocal tics, and total score) were analyzed separately. Additionally, we calculated the motor tic count per minute (tic count/minute) [17]. Due to the violations of normal distribution, we performed nonparametric tests. 

To analyze the effects of rTMS (rTMS/sham stimulation condition) on Rush values (including tic count/minute), we used the Wilcoxon signed-rank test. Rush values were compared pre- and post-rTMS in each stimulation condition. Moreover, we compared the stimulation effects (i.e., differences in the Rush values before and after rTMS) between the rTMS and sham stimulation conditions as well as between the first and second rTMS sessions (regardless of stimulation conditions). A *p*-value < 0.05 was considered statistically significant. 

For nonsignificant effects, we used Bayesian statistics to assess the evidence in favor of the null hypothesis (Bayes Factor BF_01_). According to established guidelines, a Bayes factor above 1 indicates anecdotal, above 3 substantial, and above 10 strong evidence for the null hypothesis [32,33]. Statistical analyses were run in JASP (version 0.16.2; JASP Team, 2022) [34]. 

## 3. Results

Clinical characteristics of GTS patients are given in Table 1.

There was no significant difference between the mean RMT in the rTMS (52.2 ± 8.8) and in the sham stimulation (56.5 ± 10.6) condition (*p* = 0.161). The mean stimulation intensity in the rTMS condition was 66.2 (±12.2), and in the sham condition, 22.8 (±4.3).

**Figure 2:**
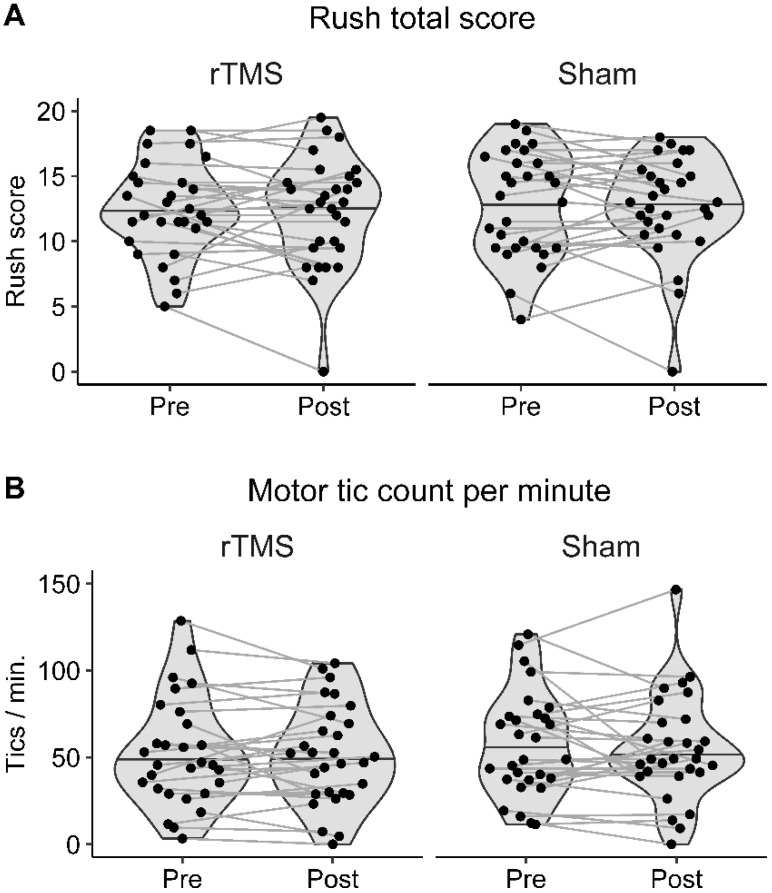
Illustration of Rush total scores and tic counts per minute. Pre- and post-rTMS (**A**) Rush total scores and (**B**) motor tic counts per minute of all participants in the rTMS and the sham stimulation condition are shown. The horizontal line in each violin plot denotes the median.

There was no significant difference between pre-rTMS Rush values in the rTMS and sham condition (*p* > 0.18 for all Rush variables and tic count/minute) or between pre- and post-rTMS Rush values in each stimulation condition (rTMS: *p* > 0.65 for all Rush variables and tic count/minute; sham: *p* > 0.28 for all Rush variables and tic count/minute). These nonsignificant effects are corroborated by a Bayesian analysis, indicating substantial evidence against a stimulation effect on the Rush total score in both stimulation conditions (rTMS: BF_01_ = 4.51; sham: BF_01_ = 4.93) and tic count/minute (rTMS: BF_01_ = 5.10; sham: BF_01_ = 4.50). This was also the case for each of the Rush subscores (BF_01_ between 2.80 and 4.87). Figure 2 shows the Rush total scores and tic counts/minute of all participants in both stimulation conditions.

Furthermore, no significant differences were found comparing stimulation effects (post- minus pre-rTMS Rush values) between rTMS and sham stimulation (*p* > 0.46 for all Rush variables and tic count/minute) and between the first and second stimulation sessions (*p* > 0.29 for all Rush variables and tic count/minute). The Bayesian analysis corroborates these null findings, indicating substantial evidence against a difference in stimulation effects between the rTMS and sham stimulation condition (Rush total score: BF_01_ = 5.00; tic count/minute: BF_01_ = 4.80) or between the first and second session (Rush total score: BF_01_ = 4.85; tic count/minute: BF_01_ = 4.13). This was also the case for each of the Rush subscores (BF_01_ between 2.36 and 4.92). 

For additional information on Rush values and statistical results, please see Supplementary Tables S1 and S2.

## 4. Discussion

To our knowledge, this is the first sham-controlled low-frequency 1 Hz rTMS study in adults with GTS examining the immediate effects of single-session 1 Hz rTMS applied to the left BA40 on tic symptoms using a blinded objective and independent video-based clinical assessment. There was no evidence for symptom reduction when comparing Rush values before and after the rTMS or sham stimulation condition. Moreover, there was no difference between both stimulation conditions. These results were corroborated by Bayesian statistics providing substantial evidence against the presence of an intervention effect. Thus, we conclude that the left BA40 is no effective target to modulate tics in adults with GTS using 1 Hz rTMS with the parameters chosen in this study. BA40 should nevertheless not be completely excluded as a target region, as there is reason to assume that repeated rTMS intervention may lead to different results, as will be discussed below.

Until now, one study reported a significant reduction in tics in GTS patients after rTMS of the parietal cortex [15]. In this study, the target region included BA40, BA7, and BA39, but no neuronavigation was used. Moreover, GTS patients received 0.5 Hz rTMS for ten consecutive days with 1200 stimuli each day [15]. Therefore, there are critical methodical differences compared to our study. It is conceivable that effects cannot be achieved with a single rTMS session as conducted in our study. Repeated intervention might have modulated tic symptoms effectively. This is plausible since in studies that have previously reported symptom reduction after rTMS, repeated stimulation was used [5,6,7]. However, these studies have limitations as only a small number of patients were included, and no sham intervention was tested. In studies including sham stimulation, no significant reduction in tic severity following rTMS, but strong placebo effects were documented [9,10,11]. Before embarking on time-consuming and logistically challenging studies in which noninvasive brain stimulation is applied over a period of many weeks, often in underpowered cohorts, there should be robust evidence for clinical efficacy in sufficiently powered short-term studies with rigorous patient assessment as in the present study. This is important to avoid the burden for patients associated with long-term assessment and repeated clinic visits. However, it is a shortcoming of this study that long-term effects at different time points were not assessed, particularly with regard to neuroplasticity induced by rTMS [4], and future studies should take this into account.

So far, tic severity was reported to be ameliorated most effectively when the SMA was used as the target region for rTMS. The reported effects were mainly based on a decrease in the YGTSS score [5,6,7,8,13,14]. In follow-up studies, the MRVRS might be helpful in evaluating tic symptoms because this allows a more objective assessment by independent, blinded raters [26]. The SMA may be a better target region than the BA40 for noninvasive brain stimulation. Still, the same misgivings as pointed out above with respect to BA40 stimulation apply to SMA protocols. Another point to consider refers to the way tic symptoms were assessed. It is conceivable that an evaluation of symptoms at home obtained by self-assessment might have been more valid since the application of rTMS can be unpleasant and somewhat stressful, thus increasing GTS symptom severity onsite [35]. However, since we did not find the effects of TMS on clinical assessment, this possibility appears unlikely.

Regarding the limitations of our study, all patients remained on their usual dose of pharmacological treatment for at least 14 days before the first rTMS session to minimize the confounding effects of medication. However, some patients received psychotropic medication that is known to affect brain excitability and, therefore, potentially also rTMS effects [28,36]. Another limitation is psychiatric comorbidity which might have affected treatment outcomes [37]. However, psychiatric comorbidities are common in GTS patients [38], and a GTS group without comorbidities does not reflect a “real-world” GTS population. The lack of a physiological marker of stimulation is a limitation of this study, which should be added in further studies. Further, it should be noted that brain activations resembling TMS-induced effects have also been documented following sham stimulation [39]. Because there was no effect following either stimulation, the latter is unlikely to have biased the results.

In summary, single-session 1 Hz rTMS versus sham stimulation of the left BA40 using rigorous and blinded patient assessment did not reduce tic symptoms in adults with GTS. Therefore, parietal low-frequency rTMS does not appear to be a useful noninvasive brain stimulation protocol in GTS using the parameters applied in this study. Further research is needed on the effects of repeated rTMS interventions, including long-term assessment.

## Figures and Tables

**Table 1:** Patient characteristics.

Subject	Age	Sex ^1^	IQ	Disease Duration (Years)	DCI ^2^ (0–100)	YGTSS ^3^ Total 120/40% RMT ^4^ (0–100)	PUTS ^5^ 120/40% RMT ^4^ (10–40)	YBOCS ^6^ (0–40)	ADHD ^7^ T-Score	
									IA ^8^	HI ^9^
1	23	M	104	16	54	n.a./58	n.a./21	0	50	49
2	21	M	106	7	39	54/40	20/18	0	47	35
3	25	M	119	9	42	38/29	20/22	11	50	45
4	46	M	111	41	76	25/12	14/14	7	51	45
5	25	M	112	19	46	15/18	9/9	0	33	47
6	44	M	115	39	100	60/44	24/26	25	79	70
7	28	F	111	14	36	53/58	22/23	0	55	38
8	47	F	123	41	48	52/29	20/19	25	79	88
9	27	M	102	11	70	41/36	26/23	20	54	54
10	20	F	117	16	78	39/47	30/30	4	43	43
11	28	M	104	22	78	60/45	20/19	10	36	42
12	39	M	121	33	76	30/53	20/23	36	56	56
13	19	M	94	16	58	61/59	16/19	17	69	38
14	32	M	117	24	59	50/47	25/21	14	42	71
15	20	F	102	12	44	29/38	23/23	21	55	38
16	24	F	96	20	72	52/44	18/19	14	43	43
17	26	F	115	21	53	60/67	30/28	20	60	56
18	20	F	112	13	58	27/48	23/22	17	53	46
19	46	M	121	35	100	36/39	19/14	12	54	55
20	50	M	89	37	36	51/28	29/26	11	58	62
21	28	F	123	25	67	48/40	14/17	8	60	46
22	49	M	110	43	27	50/53	16/15	0	42	40
23	25	F	102	23	47	31/55	19/15	0	48	46
24	18	M	110	11	35	18/21	16/21	10	57	58
25	30	F	112	24	58	75/85	23/24	23	53	59
26	27	M	89	20	91	24/20	24/14	10	36	49
27	33	F	119	27	74	47/57	27/23	15	46	45
28	28	F	108	23	80	51/50	20/18	24	88	88
29	30	F	110	25	98	66/63	32/30	19	>90	>90
Mean	30.3	-	109.4	23	62.1	44.4/44.2	21.4/20.6	12.9	54.8	53.2

^1^ M = Male, F = Female; ^2^ DCI = Diagnostic Confidence Index; ^3^ YGTSS = Yale Global Tic Severity Scale; ^4^ 120/40% RMT = stimulation intensity of 120/40% of the resting motor threshold; ^5^ PUTS = Premonitory Urge for Tics Scale; ^6^ YBOCS = Yale Brown Obsessive Compulsive Scale; ^7^ ADHD = Attention Deficit Hyperactivity Disorder; ^8^ IA = inattention; ^9^ HI = hyperactivity/impulsivity.

## Data Availability

Anonymized data can be shared by request from any qualified investigator. Data will be available for 10 years.

## Supplementary Materials

The following supporting information can be downloaded at https://www.mdpi.com/article/10.3390/biomedicines11030980/s1, Table S1: Analysis of the pre- and post-rTMS Rush values in the rTMS and sham stimulation condition in GTS patients; Table S2: Analysis of the difference of pre- and post-rTMS Rush values in the rTMS and sham stimulation condition in GTS patients.

## References

[1] American Psychiatric Association (2013). Diagnostic and Statistical Manual of Mental Disorders: DSM-5.

[2] Barker A.T., Jalinous R., Freeston I.L. (1985). Non-invasive magnetic stimulation of human motor cortex. Lancet.

[3] Dyke K., Jackson G., Jackson S. (2022). Non-invasive brain stimulation as therapy: Systematic review and recommendations with a focus on the treatment of Tourette syndrome. Exp. Brain Res..

[4] Kleimaker M., Kleimaker A., Weissbach A., Colzato L.S., Beste C., Bäumer T., Münchau A. (2020). Non-invasive brain stimulation for the treatment of Gilles de la Tourette Syndrome. Front. Neurol..

[5] Le K., Liu L., Sun M., Hu L., Xiao N. (2013). Transcranial magnetic stimulation at 1 Hertz improves clinical symptoms in children with Tourette syndrome for at least 6 months. J. Clin. Neurosci..

[6] Kwon H.J., Lim W.S., Lim M.H., Lee S.J., Hyun J.K., Chae J.H., Paik K.C. (2011). 1-Hz low frequency repetitive transcranial magnetic stimulation in children with Tourette’s syndrome. Neurosci. Lett..

[7] Mantovani A., Lisanby S.H., Pieraccini F., Ulivelli M., Castrogiovanni P., Rossi S. (2006). Repetitive transcranial magnetic stimulation (rTMS) in the treatment of obsessive-compulsive disorder (OCD) and Tourette’s syndrome (TS). Int. J. Neuropsychopharmacol..

[8] Mantovani A., Leckman J.F., Grantz H., King R.A., Sporn A.L., Lisanby S.H. (2007). Repetitive Transcranial Magnetic Stimulation of the Supplementary Motor Area in the treatment of Tourette Syndrome: Report of two cases. Clin. Neurophysiol..

[9] Münchau A., Bloem B.R., Thilo K.V., Trimble M.R., Rothwell J.C., Robertson M.M. (2002). Repetitive transcranial magnetic stimulation for Tourette syndrome. Neurology.

[10] Landeros-Weisenberger A., Mantovani A., Motlagh M.G., de Alvarenga P.G., Katsovich L., Leckman J.F., Lisanby S.H. (2015). Randomized sham controlled double-blind trial of repetitive transcranial magnetic stimulation for adults with severe Tourette syndrome. Brain Stimul..

[11] Wu S.W., Maloney T., Gilbert D.L., Dixon S.G., Horn P.S., Huddleston D.A., Eaton K., Vannest J. (2014). Functional MRI-navigated repetitive transcranial magnetic stimulation over supplementary motor area in chronic tic disorders. Brain Stimul..

[12] Orth M., Amann B., Robertson M.M., Rothwell J.C. (2005). Excitability of motor cortex inhibitory circuits in Tourette syndrome before and after single dose nicotine. Brain.

[13] Singh S., Kumar S., Kumar N., Verma R. (2018). Low-frequency repetitive transcranial magnetic stimulation for treatment of Tourette syndrome: A naturalistic study with 3 months of follow-up. Indian J. Psychol. Med..

[14] Kahl C.K., Kirton A., Pringsheim T., Croarkin P.E., Zewdie E., Swansburg R., Wrightson J., Langevin L.M., Macmaster F.P. (2021). Bilateral transcranial magnetic stimulation of the supplementary motor area in children with Tourette syndrome. Dev. Med. Child Neurol..

[15] Fu M., Wei H., Meng X., Chen H., Shang B., Chen F., Huang Z., Sun Y., Wang Y. (2021). Effects of low-frequency repetitive transcranial magnetic stimulation of the bilateral parietal cortex in patients with Tourette syndrome. Front. Neurol..

[16] Bohlhalter S., Goldfine A., Matteson S., Garraux G., Hanakawa T., Kansaku K., Wurzman R., Hallett M. (2006). Neural correlates of tic generation in Tourette syndrome: An event-related functional MRI study. Brain.

[17] Kleimaker M., Takacs A., Conte G., Onken R., Verrel J., Bäumer T., Münchau A., Beste C. (2020). Increased perception-action binding in Tourette syndrome. Brain.

[18] Sheehan D.V., Lecrubier Y., Sheehan K.H., Amorim P., Janavs J., Weiller E., Hergueta T., Baker R., Dunbar G.C. (1998). The Mini-International Neuropsychiatric Interview (M.I.N.I.): The development and validation of a structured diagnostic psychiatric interview for DSM-IV and ICD-10. J. Clin. Psychiatry.

[19] Goodman W.K., Price L.H., Rasmussen S.A., Mazure C., Fleischmann R.L., Hill C.L., Heninger G.R., Charney D.S. (1989). The Yale-Brown Obsessive Compulsive Scale. I. Development, use, and reliability. Arch. Gen. Psychiatry.

[20] Christiansen H., Hirsch O., Philipsen A., Oades R.D., Matthies S., Hebebrand J., Ueckermann J., Abdel-Hamid M., Kraemer M., Wiltfang J. (2013). German validation of the conners adult ADHD rating scale-self-report: Confirmation of factor structure in a large sample of participants with ADHD. J. Atten. Disord..

[21] Donnell A.J., Pliskin N., Holdnack J., Axelrod B., Randolph C. (2007). Rapidly-administered short forms of the Wechsler Adult Intelligence Scale-3rd edition. Arch. Clin. Neuropsychol..

[22] Leckman J.F., Riddle M.A., Hardin M.T., Ort S.I., Swartz K.L., Stevenson J., Cohen D.J. (1989). The Yale Global Tic Severity Scale: Initial testing of a clinician-rated scale of tic severity. J. Am. Acad. Child Adolesc. Psychiatry.

[23] Robertson M.M., Banerjee S., Kurlan R., Cohen D.J., Leckman J.F., McMahon W., Pauls D.L., Sandor P., van de Wetering B.J. (1999). The Tourette syndrome diagnostic confidence index: Development and clinical associations. Neurology.

[24] Woods D.W., Piacentini J., Himle M.B., Chang S. (2005). Premonitory Urge for Tics Scale (PUTS): Initial psychometric results and examination of the premonitory urge phenomenon in youths with Tic disorders. J. Dev. Behav. Pediatr..

[25] Beste C., Münchau A. (2018). Tics and Tourette syndrome—Surplus of actions rather than disorder?. Mov. Disord..

[26] Goetz C.G., Pappert E.J., Louis E.D., Raman R., Leurgans S. (1999). Advantages of a modified scoring method for the Rush Video-Based Tic Rating Scale. Mov. Disord..

[27] Paulus T., Schappert R., Bluschke A., Alvarez-Fischer D., Naumann KE R., Roessner V., Bäumer T., Beste C., Münchau A. (2021). Questioning the definition of Tourette syndrome-evidence from machine learning. Brain Commun..

[28] Paulus W., Classen J., Cohen L.G., Large C.H., Di Lazzaro V., Nitsche M., Pascual-Leone A., Rosenow F., Rothwell J.C., Ziemann U. (2008). State of the art: Pharmacologic effects on cortical excitability measures tested by transcranial magnetic stimulation. Brain Stimul..

[29] Mazziotta J., Toga A., Evans A., Fox P., Lancaster J., Zilles K., Woods R., Paus T., Simpson G., Pike B. (2001). A probabilistic atlas and reference system for the human brain: International Consortium for Brain Mapping (ICBM). Philos. Trans. R. Soc. Lond. B Biol. Sci..

[30] Takacs A., Zink N., Wolff N., Münchau A., Mückschel M., Beste C. (2020). Connecting EEG signal decomposition and response selection processes using the theory of event coding framework. Hum. Brain Mapp..

[31] Lohse A., Meder D., Nielsen S., Lund A.E., Herz D.M., Løkkegaard A., Siebner H.R. (2020). Low-frequency transcranial stimulation of pre-supplementary motor area alleviates levodopa-induced dyskinesia in Parkinson’s disease: A randomized cross-over trial. Brain Commun..

[32] Schmalz X., Biurrun Manresa J., Zhang L. (2021). What is a Bayes factor?. Psychol. Methods.

[33] Dienes Z. (2014). Using Bayes to get the most out of non-significant results. Front. Psychol..

[34] JASP Team (2022). JASP, Version 0.16.2; [Computer Software]. https://jasp-stats.org/.

[35] Robertson M.M. (2008). The prevalence and epidemiology of Gilles de la Tourette syndrome. Part 2: Tentative explanations for differing prevalence figures in GTS, including the possible effects of psychopathology, aetiology, cultural differences, and differing phenotypes. J. Psychosom. Res..

[36] Hoogendam J.M., Ramakers G.M., Di Lazzaro V. (2010). Physiology of repetitive transcranial magnetic stimulation of the human brain. Brain Stimul..

[37] Chae J.H., Nahas Z., Wassermann E., Li X., Sethuraman G., Gilbert D., Sallee F.R., George M.S. (2004). A pilot safety study of repetitive transcranial magnetic stimulation (rTMS) in Tourette’s syndrome. Cogn. Behav. Neurol..

[38] Ganos C., Münchau A., Bhatia K.P. (2014). The semiology of tics, Tourette’s, and their associations. Mov. Disord. Clin. Pract..

[39] Conde V., Tomasevic L., Akopian I., Stanek K., Saturnino G.B., Thielscher A., Bergmann T.O., Siebner H.R. (2019). The non-transcranial TMS-evoked potential is an inherent source of ambiguity in TMS-EEG studies. NeuroImage.

