# Supplementary material for: The Role of the Left Inferior Parietal Cortex in Gilles de la Tourette Syndrome—An rTMS Study
Source: Biomedicines. 2023 Mar 22;11(3):980. doi: 10.3390/biomedicines11030980 (PMC10046361; doi:10.3390/biomedicines11030980)
Supplement: Supplementary file 1 [file biomedicines-11-00980-s001.zip › biomedicines-2227401-supplementary.pdf]

Supplementary Tables

Supplementary table 1 (S1): Analysis of the pre- and post-rTMS Rush values in the rTMS and sham stimulation condition in GTS patients

Rush variable	Stimulation condition	time	Mean ± SD[1]	Wilcoxon signed rank-test[2]		
				W-value	p-value	Bayes Factor (BF_{01})
Number of body areas	verum	pre<br>post	3.33 (± 0.84)<br>3.28 (± 1.05)	51.0	0.719	4.07
	sham	pre<br>post	3.45 (± 0.71)<br>3.35 (± 0.97)	49.0	0.830	4.70
Frequency of motor tics	verum	pre<br>post	2.78 (± 1.01)<br>2.85 (± 1.10)	45.0	0.653	4.47
	sham	pre<br>post	2.97 (± 1.05)<br>2.97 (± 1.05)	54.5	0.924	4.71
Frequency of vocal tics	verum	pre<br>post	1.41 (± 1.07)<br>1.38 (± 1.18)	83.0	0.770	4.66
	sham	pre<br>post	1.55 (± 1.28)<br>1.35 (± 0.97)	159.5	0.280	2.80
Severity of motor tics	verum	pre<br>post	3.05 (± 0.70)<br>2.98 (± 0.88)	87.0	0.964	4.62
	sham	pre<br>post	3.05 (± 0.78)<br>3.07 (± 0.85)	66.0	0.617	4.49
Severity of vocal tics	verum	pre<br>post	1.90 (± 1.19)<br>1.90 (± 1.45)	126.5	1.000	4.87
	sham	pre<br>post	1.83 (± 1.29)<br>1.91 (± 1.25)	47.5	0.487	3.73
Total score	verum	pre<br>post	12.47 (± 3.57)<br>12.28 (± 4.11)	151.5	0.692	4.51
	sham	pre<br>post	12.85 (± 3.98)<br>12.67 (± 3.86)	170.5	0.840	4.93
Motor tic count per minute	verum	pre<br>post	52.41 (± 30.61)<br>51.10 (± 28.31)	212.0	0.915	5.10
	sham	pre<br>post	57.26 (± 29.60)<br>54.70 (± 30.24)	237.5	0.673	4.50

[1] Mean values and standard deviations (SD) of Rush variables, including motor tic count per minute, pre- and post-rTMS in the verum and sham stimulation condition are given.

[2] Results of Wilcoxon signed-rank test (pre- versus post-rTMS Rush value) are given.

Supplementary table 2 (S2): Analysis of the difference of pre- and post-rTMS Rush values in the rTMS and sham stimulation condition in GTS patients

Rush variable	Stimulation condition	Difference of post- and pre-rTMS Rush score: Mean ± SD[1]	Wilcoxon signed rank-test[2]		
			W-value	p-value	Bayes Factor (BF_{01})
Number of body areas	verum	-0.05 (± 0.78)	83.5	0.754	4.70
	sham	-0.10 (± 0.85)			
Frequency of motor tics	verum	0.07 (± 0.74)	85.0	0.700	4.76
	sham	0.00 (± 0.71)			
Frequency of vocal tics	verum	-0.03 (± 0.63)	162.5	0.461	3.44
	sham	-0.21 (± 0.87)			
Severity of motor tics	verum	-0.07 (± 0.76)	81.0	0.572	4.19
	sham	0.01 (± 0.58)			
Severity of vocal tics	verum	0.00 (± 0.92)	116.5	0.756	4.66
	sham	0.09 (± 0.66)			
Total score	verum	-0.19 (± 2.56)	173.0	0.959	5.00
	sham	-0.17 (± 2.41)			
Motor tic count per minute	verum	-1.30 (± 14.35)	223.0	0.915	4.80
	sham	-2.56 (± 19.15)			

[1] Mean values and standard deviations (SD) of the difference between pre- and post-rTMS Rush values (post- minus pre-rTMS Rush value) in the verum and sham stimulation condition are given.

[2] Results of Wilcoxon signed-rank test (verum versus sham stimulation condition) are given.
